# Diarrheagenic and ESBL Potential of *Escherichia coli* From Publicly Shared Common Touch Surfaces

**DOI:** 10.1002/mbo3.70125

**Published:** 2025-11-11

**Authors:** Mohammad Arif, Asma Ul Hosna, Ishrat Jahan, Md. Ashiquen Nobi, Most. Shumi Akhter Shathi, MD. Nazmul Hasan, Jayedul Hassan, S. M. Lutful Kabir

**Affiliations:** ^1^ Department of Microbiology and Hygiene Bangladesh Agricultural University Mymensingh Bangladesh; ^2^ Department of Pharmacology Bangladesh Agricultural University Mymensingh Bangladesh; ^3^ Department of Medicine Bangladesh Agricultural University Mymensingh Bangladesh

**Keywords:** antibiotic resistance, diarrheagenic *E. coli*, ESBL, frequently‐touched surfaces

## Abstract

The present study examined the virulence factors, phylogroups, antimicrobial profiles, and ESBL‐associated genes in *Escherichia coli* isolated from frequently touched surfaces (FTS) in Mymensingh Sadar, Bangladesh. A total of 105 swab samples from seven categories of publicly shared common touch surfaces were collected to assess microbial load, with *E. coli* identified through molecular assay. The isolates were screened for major diarrheagenic virulence genes and underwent phylogenetic grouping, followed by disk diffusion and double disk synergy tests to evaluate their antimicrobial resistance and ESBL potential. Microbial loads on these surfaces ranged from 6.4 to 8.56 Log_10_ CFU/cm^2^, with *E. coli* detected in 12 samples (11.43%). Among these, only two isolates carried diarrheagenic virulence factors (*ipaH* and *daaD*), classifying them as enteroinvasive and diffusely adherent *E. coli*, respectively. The isolates were distributed across all four phylogenetic groups, predominantly group A (*n* = 5; 41.7%). Notably, 10 out of 12 isolates (83.3%) were multidrug‐resistant, exhibiting complete resistance to ampicillin, followed by cefotaxime and trimethoprim/sulfamethoxazole. Additionally, the double disk synergy test confirmed four isolates (33.3%) as ESBL producers. This study highlights the potential risk of human infection with diarrheagenic *E. coli* from FTS, which may lead to serious illness and limit treatment options.

## Graphical abstract

Frequently touched surfaces (FTS) in public areas play a critical role in the transmission of infectious pathogens, including drug‐resistant bacteria. In this study, we identified multidrug‐resistant, ESBL‐producing diarrheagenic *Escherichia coli* on FTS. Our findings underscore the importance of regular microbial surveillance to monitor resistance patterns and implement effective infection control measures.

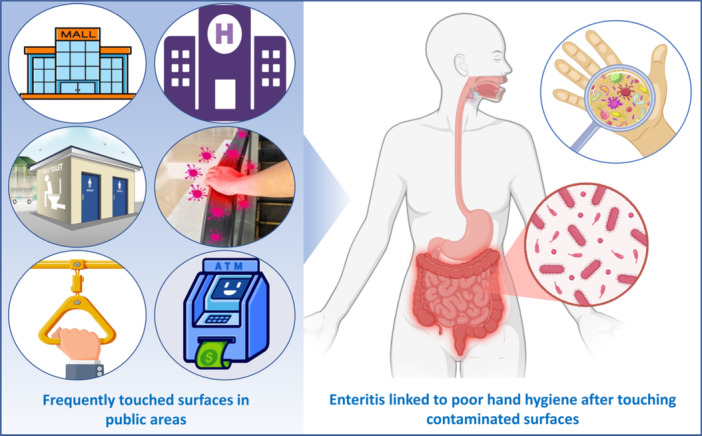

## 1 Introduction

Diarrhea is one of the most common health issues, particularly in developing nations such as Bangladesh. It is estimated that around 2 billion diarrheal cases occur every year worldwide, causing the deaths of about 1.9 million children (Rahman et al. 2020). Etiological agents behind these enteric diseases are mainly bacteria, viruses, and parasites. These infections are often connected to a lack of good hygiene practices and sanitation (Bonkoungou et al. 2021).

The Global Burden of Disease (GBD) study (2015) assessed the etiology of the deaths resulting from diarrheal disease in Bangladesh, where *Escherichia coli* was revealed as one of the leading causal agents (Troeger et al. 2017). In the intestinal tracts of both humans and animals, *E. coli* is a vital component of the normal microflora. Currently, diarrheagenic *E. coli* (DEC) is distributed into six classes: enteropathogenic (EPEC), enterohemorrhagic (EHEC) or Shiga toxin‐producing *E. coli* (STEC), enterotoxigenic (ETEC), enteroinvasive (EIEC), enteroaggregative (EAEC), and diffusely adherent *E. coli* (DAEC) (Hossain et al. 2021). Besides, *E. coli*, which produces cytolethal‐distending toxins, has also been observed to be responsible for childhood watery diarrhea (Hossain et al. 2021). All these pathotypes have already been reported in Bangladesh from various sources (Sahl et al. 2017; Parvej et al. 2020).

A source of pathogens, a transient environmental reservoir of the pathogenic microorganisms (frequently touched surfaces [FTS]), and a target population at increased risk for infection comprise the fundamental elements of pathogen transfer in homes and communities (Scott 2013). Environmental surfaces are an indispensable part of indirect transmission. The reservoir sheds pathogens, which are transmitted back to other susceptible hosts by means of hand contact, cross‐contaminated foods, or other surfaces. Moreover, FTS are believed to have a significant role in nosocomial transmission (Suleyman et al. 2018). Both laboratory and field studies established the survival of pathogens on FTS (Hossain et al. 2021; Katzenberger et al. 2021). The survival length of pathogens on these surfaces depends mainly on bacterial species and bioburden, the potential of biofilm formation, surface material type, pH, availability of moisture and nutrients, ambient temperature and humidity, the magnitude of UV radiation, and so on (Hossain et al. 2021; Katzenberger et al. 2021). Although many Gram‐positive pathogens were found to have longer survivability (a few weeks) on inanimate surfaces, most Gram‐negative pathogens, including *E. coli*, were found to survive from a few hours to days (Katzenberger et al. 2021).

Therefore, based on available information, it would not seem irrational to expect that common touch surfaces may contribute similarly to pathogen transmission in residential and communal settings. Consequently, Cobrado et al. (2017) documented some frequently involved bacteria, such as methicillin‐resistant *Staphylococcus aureus* (MRSA), vancomycin‐resistant enterococci (VRE), *Clostridium difficile*, and multidrug‐resistant (MDR) Gram‐negative bacilli, including Enterobacteriaceae, that may survive on healthcare‐associated equipment and environmental surfaces. So far, the potential risk of nosocomial transmission has been better understood. Still, defining other frequently shared common surfaces in public areas regarding pathogen transmission is more time‐demanding. Hossain et al. (2021) described the environmental transmission of diarrhea‐causing pathogens to humans. According to the Worldwide Outbreak Database (August 2020), out of 3632 nosocomial outbreak reports, *E. coli* shared about 2.4% (86/3632) (Katzenberger et al. 2021). Moreover, Warnes et al. (2012) reported that *E. coli* harboring extended‐spectrum‐β‐lactamase (ESBL) bla_CTX‐M‐15_ exhibited prolonged survival on stainless steel. Hence, the potential for the survival of DEC on common touch surfaces might be detrimental to public health.

Previous studies have identified hospital and community wastewater, livestock farms, and slaughterhouses as major sources of antimicrobial‐resistant pathogens that are disseminated into the environment (Homeier‐Bachmann et al. 2021; Moniruzzaman et al. 2023). Hospitals, in particular, pose a high risk due to overcrowding, insufficient bed capacity, and patients being placed on floors and hallways, all of which contribute to increased contamination of FTS. Inadequate sanitation practices further exacerbate this issue, heightening the risk of resistant bacterial transmission and hospital‐acquired infections (Moniruzzaman et al. 2023). Beyond healthcare settings, public and even household washroom surfaces serve as potential reservoirs of pathogens (Chang et al. 2022). Poor hand hygiene following restroom use can lead to contamination of human hands, which in turn facilitates the spread of pathogens to FTS in public spaces. Additionally, inadequate sanitation and improper cleaning and disinfection of FTS can accelerate pathogen transmission, posing a significant public health concern.

Despite the potential for pathogen transmission through FTS, data on DEC contamination on publicly shared touch surfaces remain limited. Although numerous studies have reported the presence of *E. coli* on various FTS, only a few have assessed its potential to cause diarrhea. Patel et al. (2023) reported *E. coli* from the trolleys and basket handles in shopping malls in Zambia. Dawodu and Akanbi (2021) isolated *E. coli* along with other enteric and skin commensal bacteria from the automated teller machines. Public restrooms, with their warm and humid conditions, provide a favorable environment for microbial survival. Previous studies have identified public restrooms as reservoirs for various pathogens due to inadequate cleanliness and poor hand hygiene, with *E. coli* frequently recovered from different surface samples (Kouadri 2020; Chang et al. 2022). Ghanem and Haddadin (2018) investigated *E. coli* contamination in both hospital and household settings, reporting a prevalence of 4.9%, of which 61.9% were identified as ESBL producers. ESBL‐producing *E. coli* is a MDR pathogen responsible for severe hospital‐ and community‐acquired infections, particularly in lower‐middle‐income countries with poor sanitation and hygiene (Moniruzzaman et al. 2023). Compared to non‐ESBL *E. coli*, these infections are associated with higher mortality, morbidity, prolonged hospital stays, and increased healthcare costs. ESBL *E. coli* is resistant to multiple antibiotics commonly used for *E. coli* infections, often rendering treatment ineffective. The spread of ESBL *E. coli* is largely driven by horizontal gene transfer, allowing pathogenic *E. coli* strains to acquire resistance genes and further enhance their antimicrobial resistance (AMR) (Moniruzzaman et al. 2023).

In Bangladesh, limited studies have focused on the phenotypic and molecular characterization of ESBL‐producing *E. coli*, particularly in environmental isolates (Hossain et al. 2021; Moniruzzaman et al. 2023). Moniruzzaman et al. (2023) suggested a widespread prevalence of ESBL *E. coli* in hospital patients of Bangladesh, with 26% ESBL‐producing *E. coli* from various environmental and clinical specimens. However, none of these isolates exhibited diarrheagenic potential. In contrast, Hossain et al. (2021) reported the diarrheagenic pathotypes of *E. coli* from frequently touched household kitchen and restroom surfaces without assessing any environmental surfaces from public areas. However, to the best of our knowledge, no study to date has investigated the presence of ESBL‐producing and DEC on frequently touched inanimate surfaces in various indoor and outdoor public areas, which might act as reservoirs for microorganisms, including MDR bacteria, facilitating their spread through hand contact. Therefore, the present study was designed to isolate DEC from several frequently touched indoor and outdoor surfaces in public areas, discriminate between their pathotypes, and elucidate their AMR and ESBL‐producing ability. The information generated from this study will help raise public awareness about personal hygiene and environmental hygiene to reduce transmission of pathogens and develop baseline data that will aid in selecting proper therapeutic interventions to control enteric pathogens like *E. coli*.

## 2 Materials and Methods

### 2.1 Study Area

The present study was carried out at different locations in Mymensingh Sadar Upazila (located between 24°38′ and 24°54′ North latitudes and 90°11′ and 90°30′ East longitudes) of the Mymensingh district of Bangladesh. Mymensingh Sadar is the most populous upazila (population density of 1736 per km^2^) of Mymensingh district, having an area of 388.45 km^2^. Numerous educational institutions, public hospitals, shopping malls, administrative offices, mosques, bus and train terminals, and other facilities are spread around the city.

### 2.2 Sample Collection

A total of 105 surface swab samples were collected from various public locations across Mymensingh Sadar between March and August 2022, following the protocol described by Odoyo et al. (2021). Seven distinct surface types were selected, with 15 samples collected from each type, resulting in 105 samples in total. The sampled surfaces included staircase railings, ATM keyboards, public transport grab rails, elevator buttons, public restroom doorknobs, restroom flush buttons, and restroom tap water knobs (Table 1). These samples were obtained from high‐traffic areas such as hospitals, shopping malls, government offices, academic institutions, mosques, and bus stations. No specific proportional distribution was maintained among the collection sites.

**Table 1:** Occurrence of diarrheagenic *Escherichia coli* (DEC) from surface samples.

Types of samples collected	No. of samples (*n*)	No. (%) of positive *E. coli*	*p* value^b^	Diarrheagenic *E. coli*		
				EIEC (%)^a^	DAEC (%)^a^	Total (%)^a^
Staircase railings (SR)	15	0	0.002	0	0	0
ATM keyboards (ATM)	15	0		0	0	0
Public transport grab rails (PTG)	15	1 (6.67)		0	0	0
Elevator buttons (EB)	15	1 (6.67)		0	1 (6.67)	1 (6.67)
Public restroom doorknobs (TDK)	15	2 (13.33)		0	0	0
Public restroom flush buttons (TFB)	15	1 (6.67)		0	0	0
Public restroom tap water knobs (TTWK)	15	7 (46.67)		1 (6.67)	0	1 (6.67)
Total	105	12 (11.43)		1 (0.95)	1 (0.95)	2 (1.9)

Abbreviations: DAEC, diffusely adherent *E. coli*; EIEC, enteroinvasive *E. coli*; *n*, number of samples to be tested.

^a^ Percentage was calculated in each surface sample type.

^b^ Fisher's exact test was carried out to determine the significant difference in the occurrence of *E. coli* isolates. A *p*‐value less than 0.05 (*p* < 0.05) was regarded as a significant difference.

Before sample collection, verbal consent was obtained from the relevant authorities at each location—for instance, from facility managers (e.g., public banks) for ATM keyboards, drivers of public transport vehicles for grab rails, and responsible personnel from public offices, restaurants, shopping malls, mosques, and hospitals for staircase railings, elevator buttons, and restroom facilities. Since only environmental surface swabs were collected from public or institutional areas, without involving human participants or collecting personal data, written consent was not deemed necessary.

To standardize the sampling area, a 5 cm^2^ surface area was demarcated for each swab. For surfaces smaller than 5 cm^2^, such as elevator buttons, public restroom doorknobs, flush buttons, and tap water knobs, the entire surface was swabbed. Pre‐moistened sterile cotton swabs soaked in phosphate‐buffered saline (PBS) were used to sample the defined area by gently wiping and rotating the swab to ensure full contact of all sides of the swab tip with the surface. Each surface was covered by a single swab. Following collection, the swabs were immediately placed into sterile Eppendorf tubes containing 1 mL of PBS, labeled appropriately, and transported to the laboratory within a few hours under cooled conditions to maintain sample integrity.

### 2.3 Bacterial Enumeration From the Surface Samples

Samples underwent immediate processing to assess the viable bacterial load of the respective surfaces according to the procedure described by Odoyo et al. (2021) with slight modifications. Initially, swab samples in 1 mL PBS were vortexed properly to get a homogenized solution. After that, using sterile PBS, 10‐fold serial dilutions of the samples were made. 100 µL of the sample from 1:100 to 1:100,000 dilution was inoculated on plate count agar (HiMedia, India). A sterile glass spreader disseminated the inoculum evenly over the surface of the culture plate. For each dilution, three plates were used to get an average colony count from a specific dilution. Plates were then inoculated at 37°C for 24 h. After incubation, a specific dilution showing 30–300 colonies per plate was chosen, and the average colony count of that particular dilution was noted. Subsequently, the bacterial load of each surface sample was quantified by calculating the colony‐forming units per milliliter (CFU/mL), which corresponded to the number of CFUs recovered from a 5 cm^2^ area. The results were then recalculated and expressed as CFUs per square centimeter (CFU/cm^2^) to accurately represent the surface contamination level.

### 2.4 Isolation and Identification of *E. coli*

*E. coli* was isolated from the surface samples following standard procedures described by Atnafie et al. (2017) with slight modifications. Briefly, 500 µL was transferred from the original swab suspension to the Tryptone Soya Broth (TSB broth, HiMedia, India) and incubated overnight at 37°C for enrichment. Enriched samples were then streaked onto MacConkey agar (Merck, Germany), followed by overnight incubation at 37°C. Bacterial colonies supposed to be lactose‐fermenting were further studied by Gram staining. Then, *E. coli* suspected colonies were cultured on Eosin Methylene Blue (EMB, Merck, Germany) and MacConkey Sorbitol Agar (HiMedia, India) and incubated for 24 h. Green colonies having a metallic sheen from the EMB agar were selected as typical *E. coli* colonies. Possibly colorless colonies in MacConkey agar plates with sorbitol indicated the O157:H7 strain, as they do not ferment sorbitol (Bautista‐Trujillo et al. 2020). Additionally, *E. coli* colonies were analyzed by a few biochemical tests (glucose and lactose fermentation, indole, oxidase, and catalase tests). *E. coli* isolates were preserved at −20°C in Brain Heart Infusion (BHI) broth (Merck, Germany) with 15% (V/V) glycerol for further characterization.

### 2.5 Molecular Detection of *E. coli*

The genomic DNA extraction of the isolates was conducted through the boiling method, following the procedure described earlier (Bag et al. 2021). *malB* gene‐based PCR (polymerase chain reaction) was performed from the extracted genomic DNA for molecular detection of *E. coli*, emulating the protocol reported previously (Wang et al. 1996). In summary, a 20 µL volume of PCR reaction mixture was prepared by mixing 10 µL of 2X GoTaq G2 Master Mix (Promega, USA) with 10 pmol of forward and reverse primers (Supporting Information S2: Table S1) and 2 µL of genomic DNA. The PCR result was compared with the positive control *E. coli* ATCC 25922. An initial denaturation at 95°C for 5 min, followed by 30 cycles of 94°C for 45 s, 52°C for 45 s, and 72°C for 60 s, and a final extension for 5 min at 72°C, was employed for the amplification of the targeted gene using a thermal cycler (ASTEC 482, Japan). Each PCR product generated in this study was placed under 1.5% agarose gel electrophoresis, stained with ethidium bromide solution, and finally imaged under UV lights (Uvsolo TS, Biometra).

### 2.6 Molecular Identification of Major Virulence Genes of DEC

All *E. coli* isolates in this study were examined for their essential virulence genes to designate them as DEC. DEC pathotypes were determined by investigating the virulence genes using single PCR reactions: *eaeA* gene for both typical (tEPEC) and atypical enteropathogenic *E. coli* (aEPEC) (Fallah et al. 2021), *bfpA* gene for tEPEC (Fallah et al. 2021), *stx1* and *stx2* genes for enterohemorrhagic *E. coli* (EHEC/STEC) (Talukdar et al. 2013), *ipaH* and *virF* genes for EIEC (Canizalez‐Roman et al. 2013), LT and ST genes for ETEC (Girard et al. 2020; Sobhy et al. 2020), pCVD432 and *aggR* genes for EAEC (Fallah et al. 2021), and *daaD* gene for DAEC (Fallah et al. 2021). Along with the diarrheagenic virulence determinants, the *rfbE* gene, which produces lipopolysaccharide O in *E. coli*, was also screened to detect STEC O157 (Paton and Paton 1998). For PCR, a 20 µL reaction mixture was prepared in the same way described earlier, and the amplification was carried out in a thermal cycler (ASTEC, Japan) with the thermal profiles listed in Supporting Information S2: Table S1. Primer sequences of each PCR reaction are also listed in Supporting Information S2: Table S1.

### 2.7 Phylogenetic Grouping of *E. coli* Isolates

The distribution of four major *E. coli* phylogenetic groups (A, B1, B2, and D) was determined using the triplex PCR method described by Clermont et al. (2000) based on the presence of *chuA* and *yjaA* genes and a DNA fragment TSPE4.C2. Primer sequences and thermal profiles of each PCR reaction for detecting phylogroups are mentioned in Supporting Information S2: Table S1. The targeted genes were amplified in a thermal cycler (ASTEC, Japan) with 20 µL PCR reaction mixtures, as described earlier.

### 2.8 Detection of ESBL‑Producing *E. coli* and Antimicrobial Susceptibility Testing

Resistance patterns of the *E. coli* isolates against numerous antimicrobial agents were revealed by the Kirby‐Bauer disk diffusion test (Bauer et al. 1966), and the results were interpreted as susceptible, intermediate, and resistant compared with the standard guidelines of the Clinical and Laboratory Standards Institute (Clinical and Laboratory Standards Institute [CLSI] 2018). A total of 13 antimicrobials from 10 different antimicrobial classes, frequently applied in human and veterinary medicine in Bangladesh, were tested in the current study. Commercially available antibiotic disks (Oxoid, UK), such as aminoglycosides (neomycin – 30 µg, gentamicin – 10 µg), cephalosporins (ceftazidime – 30 µg, cefotaxime – 30 µg), fluoroquinolones (ciprofloxacin – 5 µg), macrolides (azithromycin – 15 µg), penicillins (ampicillin – 10 µg, amoxycillin/clavulanic acid – 30 µg), phenicols (chloramphenicol – 30 µg), carbapenems (imipenem – 10 µg), nitrofurans (nitrofurantoin – 300 µg), sulfonamides (trimethoprim/sulfamethoxazole – 25 µg), and tetracyclines (tetracycline – 30 µg) were used to reveal the resistance profiles of the isolates. In addition, isolates with resistance to three or more different antibiotic classes were referred to as MDR (Rafailidis and Kofteridis 2022). Results were validated with the control strain *E. coli* ATCC 25922.

Phenotypic identification of ESBL‐producing *E. coli* was performed using the double‐disk synergy test (DDST) in accordance with CLSI guidelines (Clinical and Laboratory Standards Institute [CLSI] 2018). Briefly, fresh bacterial cultures were adjusted to the 0.5 McFarland turbidity standard and uniformly inoculated onto Mueller‐Hinton agar plates (HiMedia, India) using sterile swabs. An amoxicillin‐clavulanic acid disk (20/10 µg) was placed at the center of the agar plate. Two other antibiotic disks—cefotaxime (30 µg) and ceftazidime (30 µg)—were positioned at a distance of 30 mm (center to center) from the central disk. The plates were then incubated at 37°C for 18–24 h. An isolate was considered ESBL‐producing if it exhibited resistance to ceftazidime (inhibition zone ≤ 17 mm) and cefotaxime (inhibition zone ≤ 22 mm), along with a ≥  5 mm increase in the zone of inhibition of either antibiotic disk when positioned adjacent to the amoxicillin‐clavulanic acid disk, indicating synergistic activity.

### 2.9 Multiple Antimicrobial Resistance Index (MARI)

The MARI for *E. coli* isolates was calculated using the mathematical equation as described by Msolo et al. (2020), which is expressed as

MARindex=(Numberofantibioticstowhichresistanceoccurred/Sumofantibioticstowhichanindividualisolatewastested).

### 2.10 Molecular Characterization of ESBL‐Producing *E. coli*

All the *E. coli*, including the phenotypically identified ESBL‐producing isolates, were further examined by PCR to detect ESBL‐related genes (ESBL‐associated genes, including *bla*_*TEM‐1*_, *bla*_*SHV*_, and *bla*_*CTX‐M*_), as described earlier (Chen et al. 2004). Primer sequences and thermal profiles associated with different β‐lactamase genes are documented in Supporting Information S2: Table S1. The PCR reaction volume was adjusted to 20 µL, and amplification was performed in a thermal cycler (ASTEC, Japan).

### 2.11 Statistical Analysis

#### 2.11.1 Descriptive Analysis

The total bacterial load from each of the seven surface types was analyzed using one‐way analysis of variance (ANOVA) to compare the means among groups. To identify specific group differences, Tukey's Honest Significant Difference post hoc test was applied. The results were expressed as mean ± standard error of the mean (SEM) for each group. In contrast, differences in the occurrence of *E. coli* across seven different surface sample types were analyzed using Fisher's exact test, suitable for small sample sizes and low expected frequencies, using the Statistical Package for the Social Sciences (SPSS) software (version 22.0, IBM Corp., Armonk, NY, USA).

#### 2.11.2 Bivariate Analysis

A Pearson correlation analysis was performed using SPSS (version 22.0) to assess the relationships between resistance to individual antibiotics in *E. coli* isolates, while a Spearman correlation was used to evaluate associations among ESBL‐associated genes.

#### 2.11.3 Logistic Regression

Binary logistic regression analysis was conducted using SPSS (version 22.0) to evaluate associations between the presence of ESBL‐associated genes (independent variable) and two sets of dependent variables: (i) AMR patterns and (ii) *E. coli* pathotypes (DEC types). The analysis followed the “Enter” method. For the purpose of regression modeling, isolates were categorized as ESBL gene‐positive (coded as 1) or ESBL gene‐negative (coded as 0, reference category). Antimicrobial susceptibility data were dichotomized: intermediate and resistant results were combined and classified as “resistant,” while susceptible isolates were categorized as “susceptible.” Similarly, *E. coli* isolates were classified according to the presence or absence of DEC pathotypes. Odds ratios with 95% confidence intervals were reported to quantify the strength of association. A *p*‐value < 0.05 was considered statistically significant in all analyses.

## 3 Results

### 3.1 Bacterial Load and Occurrence of *E. coli*

**Figure 1:**
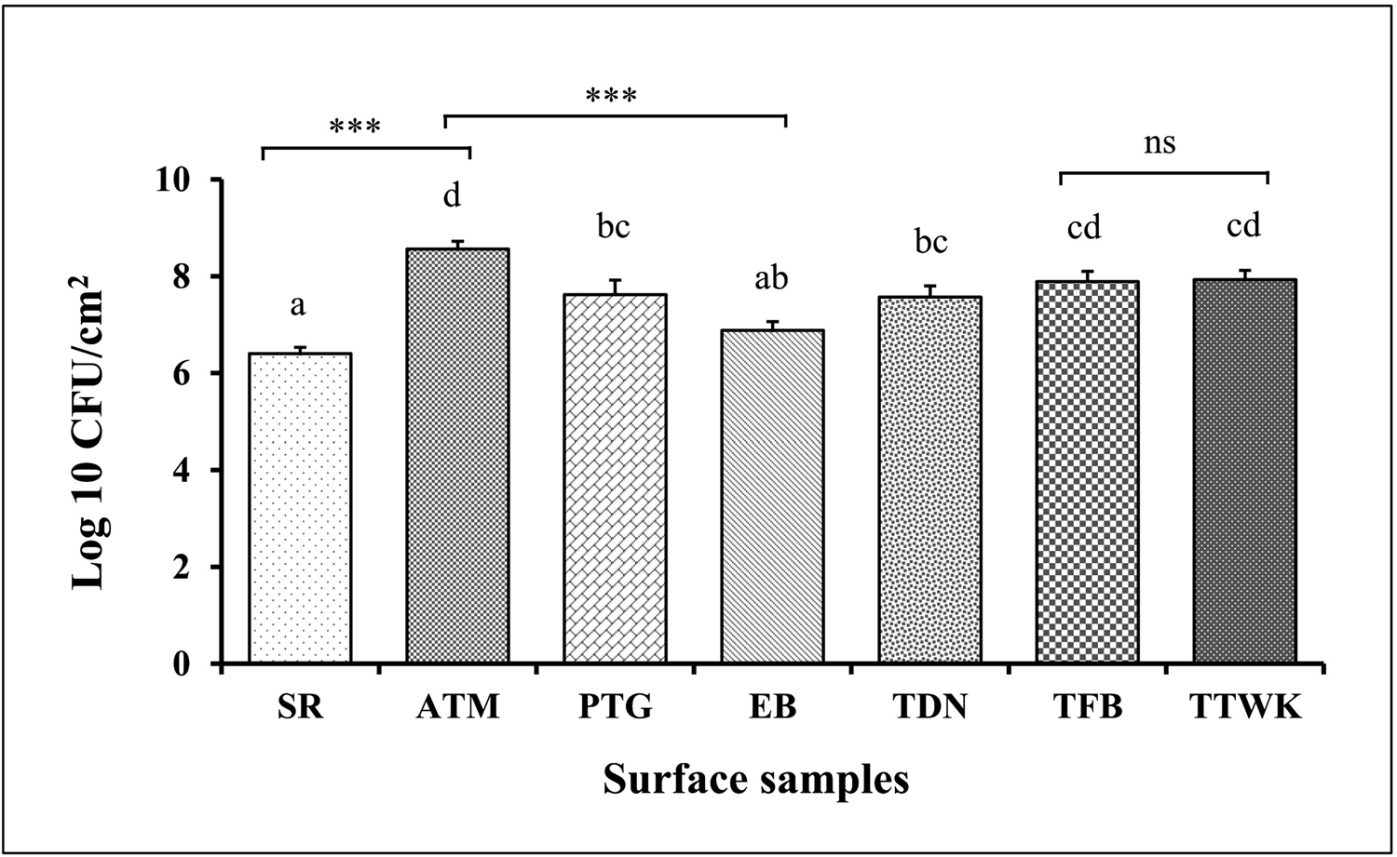
Assessment of bacterial load on different surface samples. Each bar indicates the average mean ± SEM value of the surface samples. One‐way ANOVA was conducted to assess significant differences in the average bacterial load across different surface types. ATM, ATM keyboards; EB, elevator buttons; PTG, public transport grab rails; SR, staircase railings; TDN, public restroom doorknobs; TFB, public restroom flush buttons; TTWK, public restroom tap water knobs. ^a,b,c,d^Bars with different alphabets differ significantly (*p* < 0.05); *** denotes highly significant data (*p* < 0.001); ns = non‐significant (*p* > 0.05).

Almost every surface sample examined in this study was contaminated with a large number of bacteria. The population density of viable bacteria recorded from the surface samples ranged from 6.4 to 8.56 Log10 CFU/cm^2^. The highest average bacterial load was identified from the keyboard of ATM surfaces (8.56 Log10 CFU/cm^2^), which significantly varied (*p* < 0.05) from the bacterial load recorded in staircase railing (6.40 Log10 CFU/cm^2^) and elevator button (6.88 Log10 CFU/cm^2^) samples. The lowest average bacterial load was recorded from the staircase railing samples (6.40 Log10 CFU/cm^2^) (Figure 1).

**Figure 2:**
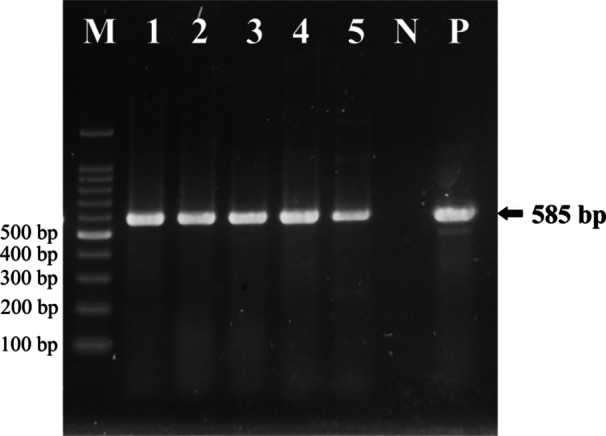
Representative photograph of PCR targeting the *Escherichia coli*‐specific *malB* gene. Lanes: 1–5: suspected *E. coli* isolates; Lane N: Negative control without DNA samples; Lane P: *E. coli* ATCC 25922 (Positive control); Lane M: 100 bp DNA ladder, Promega. The electrophoresis was carried out using a 1.5% agarose gel (Promega) at 100 V for 25 min in 1× TAE buffer.

All surface swab samples (*n* = 105) were subjected to standard cultural and biochemical methods for the isolation and preliminary identification of *E. coli*, resulting in the successful isolation of *E. coli* from 12 samples (11.43%). None of the isolates exhibited typical growth on MacConkey Sorbitol Agar, suggesting the absence of the *E. coli* O157:H7 serotype. All 12 culture‐positive isolates were further confirmed as *E. coli* by PCR amplification targeting the *malB* gene (Table 1; Figure 2). The occurrence of *E. coli* varied significantly (*p* < 0.05) among the different sample types, with the highest prevalence observed in public restroom tap water knobs (*n* = 7, 46.67%). In contrast, no *E. coli* was detected in samples collected from staircase railings and ATM keyboards (*n* = 0; 0.0%).

### 3.2 Virulence Determinants and Phylogenetic Grouping of *E. coli* Isolates

None of the 12 *E. coli* isolates were positive for *rfbE* O157, *stx1*, *stx2*, *eaeA*, *bfpA*, *virF*, ST, LT, *aggR*, and pCVD432 (Supporting Information S2: Table S2). Only two isolates carried two distinct virulence genes, *ipaH* and *daaD*, and were thus identified as EIEC and EAEC, respectively (Table 1; Supporting Information S1: Figure S1). Simultaneously, PCR targeting *chuA*, *yjaA*, and DNA fragment TspE4.C2 was carried out for the phylogenetic grouping of isolated *E. coli* (Supporting Information S1: Figure S2). PCR results, when compared to the reference method, revealed five isolates in group A (41.7%), two in group B1 (16.7%), four in group B2 (33.3%), and one isolate in group D (8.3%). Out of two DEC pathotypes, EIEC belonged to group B1 and DAEC belonged to group D according to the typing procedure employed (Supporting Information S2: Table S2).

### 3.3 Antimicrobial Susceptibility Patterns of the Isolated *E. coli*

**Figure 3:**
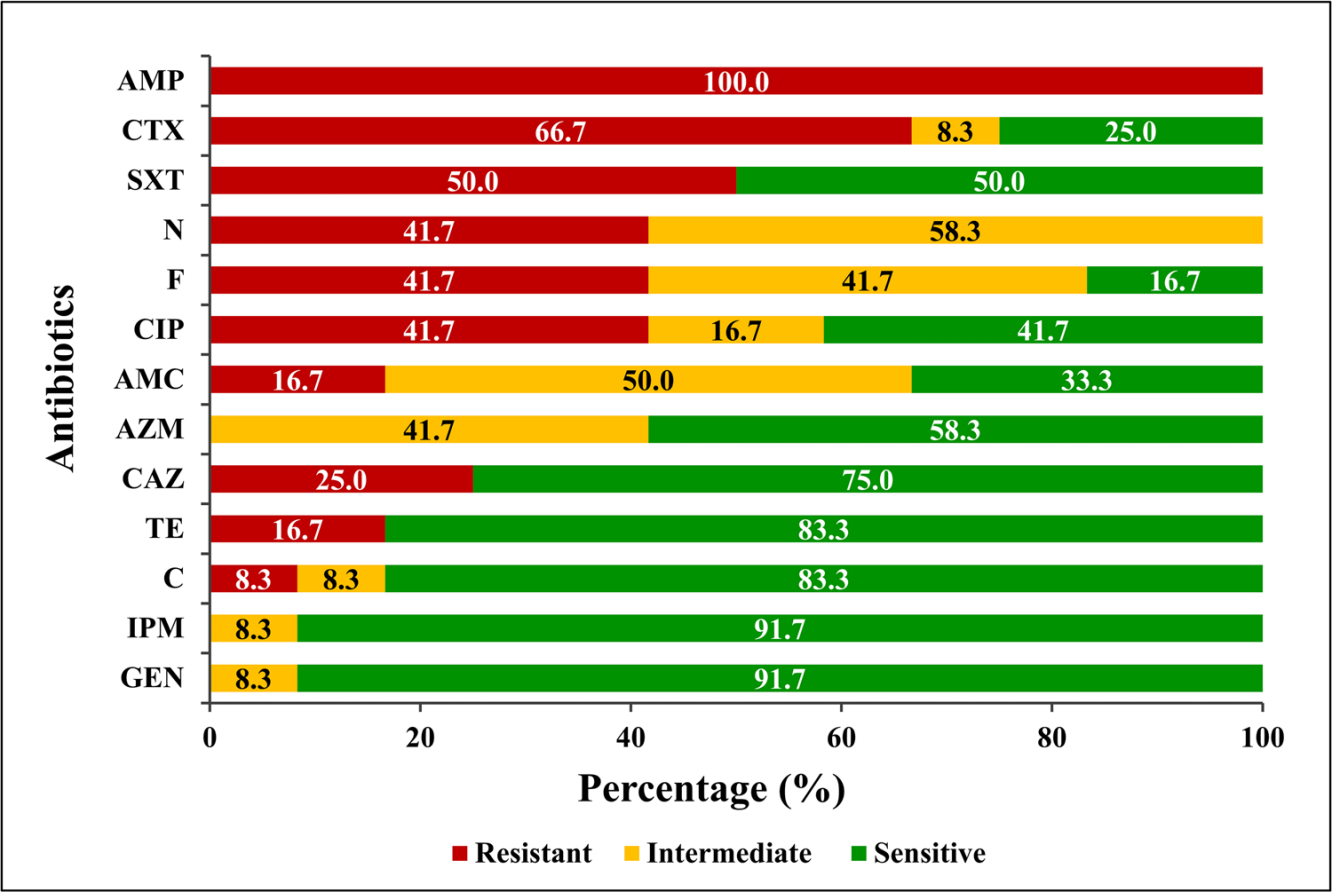
Antimicrobial susceptibility profile of the *Escherichia coli* isolates. AMC, amoxycillin/clavulanic acid (30 µg); AMP, ampicillin (µg) 10 µg; AZM, azithromycin (15 µg); C, chloramphenicol (30 µg); CAZ, ceftazidime (30 µg); CIP, ciprofloxacin (5 µg); CTX, cefotaxime (30 µg); F, nitrofurantoin (300 µg); GEN, gentamicin (10 µg); IPM, imipenem (10 µg); N, neomycin (30 µg); SXT, trimethoprim/sulfamethoxazole (25 µg); TE, tetracycline (30 µg).

The antimicrobial susceptibility of 12 *E. coli* isolates was determined against 13 antibiotics from 10 different antibiotic classes. All the *E. coli* isolates recovered in this study showed varying percentages of AMR, with the highest resistance to ampicillin (*n* = 12; 100%), followed by cefotaxime (*n* = 8; 66.7%) and trimethoprim/sulfamethoxazole (*n* = 6; 50%) (Figure 3). However, almost all the isolates were sensitive to gentamicin and imipenem. Each of the isolates exhibited resistance to at least one of the β‐lactam antibiotics (Supporting Information S2: Table S2). Out of 12 isolates, 3 (25%) were found to be resistant to all 3 β‐lactam antibiotics (ampicillin, cefotaxime, and ceftazidime) examined in this study, where 1 isolate belonged to the DAEC pathotype.

**Figure 4:**
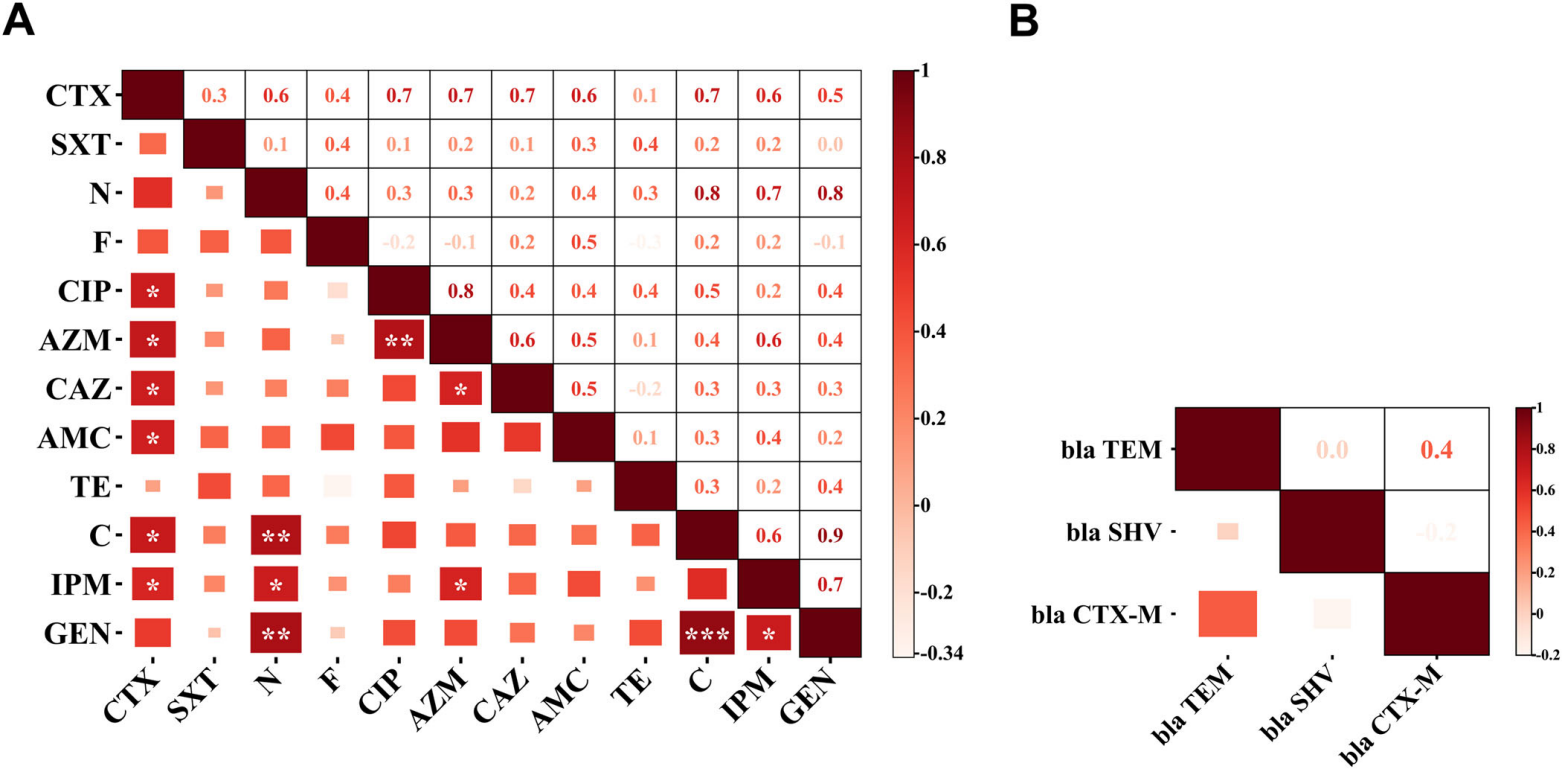
Representation of the correlation matrix of antimicrobial resistance of *Escherichia coli* isolates. (A) Pearson correlation matrix analysis of the phenotypic resistances of different antimicrobials. (B) Spearman correlation matrix analysis of the ESBL‐associated genes detected from the *E. coli* isolates. *Correlation is significant at the 0.05 level (two‐tailed). *p < 0.05; **p < 0.01; ***p < 0.001. In this figure, larger rectangle sizes and stronger color intensities indicate higher correlation coefficient values, representing stronger associations between pairs of antibiotics. AMC, amoxycillin/clavulanic acid (30 µg); AZM, azithromycin (15 µg); C, chloramphenicol (30 µg); CAZ, ceftazidime (30 µg); CIP, ciprofloxacin (5 µg); CTX, cefotaxime (30 µg); F, nitrofurantoin (300 µg); N, neomycin (30 µg); SXT, trimethoprim/sulfamethoxazole (25 µg); TE, tetracycline (30 µg).

A bivariate analysis was performed to find out the correlation among the phenotypic resistances. A highly strong positive and significant correlation was observed between chloramphenicol and gentamicin (Pearson correlation coefficient, *ρ* = 0.87; *p* < 0.001). Additionally, strong positive and significant correlations were detected between cefotaxime and ciprofloxacin, cefotaxime and azithromycin, cefotaxime and ceftazidime, cefotaxime and chloramphenicol, neomycin and chloramphenicol, neomycin and imipenem, neomycin and gentamicin, ciprofloxacin and azithromycin, and imipenem and gentamicin (Figure 4A).

### 3.4 MDR and MARI

The present study identified 10 (83.3%) MDR isolates with 10 different MDR patterns among the 12 *E. coli* isolates. Among the MDR, one isolate revealed resistance against eight antimicrobial classes, four against five classes, three against four classes, and the remaining two were resistant to three antimicrobial classes (Table 2). The median MARI of the 12 *E. coli* isolates was 0.34 (range: 0.08–0.62), with 10 (83.3%) of the isolates having MARI > 0.2 (Supporting Information S2: Table S2).

**Table 2:** Multidrug resistance (MDR) and multiple antibiotic resistance index (MARI) profiles of the *Escherichia coli* isolates.

Isolate no.	Resistance phenotypes	No. of antibiotics	No. of antimicrobial classes	No. (%) of MDR isolates (*n* = 12)	Overall no. (%) of MDR isolates (*n* = 12)	MARI
1	AMP‐CTX‐SXT‐N‐CIP‐AZM‐TE‐C	8	8	1 (8.33)	10 (83.33)	0.62
2	AMP‐CTX‐SXT‐CIP‐AZM‐CAZ	6	5	1 (8.33)		0.46
3	AMP‐CTX‐SXT‐N‐F‐CAZ	6	5	1 (8.33)		0.46
4	AMP‐CTX‐CIP‐AZM‐CAZ‐AMC	6	4	1 (8.33)		0.46
5	AMP‐CTX‐SXT‐F‐AZM	5	5	1 (8.33)		0.38
6	AMP‐CTX‐N‐F‐CIP	5	5	1 (8.33)		0.38
7	AMP‐SXT‐CIP‐TE	4	4	1 (8.33)		0.31
8	AMP‐CTX‐N‐AZM	4	4	1 (8.33)		0.31
9	AMP‐SXT‐F‐AMC	4	3	1 (8.33)		0.31
10	AMP‐CTX‐F	3	3	1 (8.33)		0.23
11	AMP	1	1	—		0.08
12	AMP‐N	2	2	—		0.15

Abbreviations: AMC, amoxycillin/clavulanic acid (30 µg); AMP, ampicillin (µg) 10 µg; AZM, azithromycin (15 µg); C, chloramphenicol (30 µg); CAZ, ceftazidime (30 µg); CIP, ciprofloxacin (5 µg); CTX, cefotaxime (30 µg); F, nitrofurantoin (300 µg); N, Neomycin (30 µg); SXT, trimethoprim/sulfamethoxazole (25 µg); TE, tetracycline (30 µg).

### 3.5 Phenotypic and Genotypic Characterization of ESBL‐Producing *E. coli*

**Figure 5:**
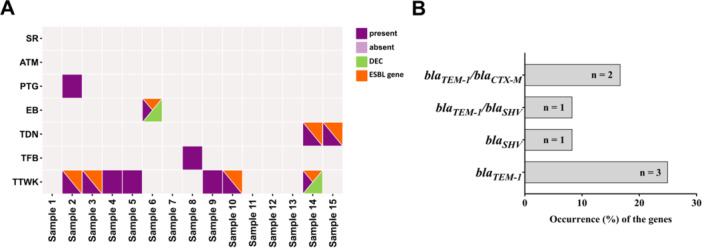
Distribution of diarrheagenic *Escherichia coli* and identification of ESBL genes in various FTS samples. (A) Overview of virulence and resistance traits among *E. coli*‐positive surface samples (*n* = 12). In this figure, deep purple color indicates the presence of *E. coli* in the respective samples, while light purple signifies its absence. Green indicates diarrheagenic *E. coli* pathotype, whereas orange represents the presence of at least one of the tested ESBL‐associated genes. (B) Occurrence of ESBL genes (*bla*_*TEM‐1*_, *bla*_*SHV*_, and *bla*_*CTX‐M*_) among 12 *E. coli* positive FTS samples. ATM, ATM keyboards; EB, elevator buttons; PTG, public transport grab rails; SR, staircase railing; TDN, public restroom doorknobs; TFB, public restroom flush buttons; TTWK, public restroom tap water knobs.

The occurrence of ESBL‐producing *E. coli* was determined using the DDST. Among 12 *E. coli* isolates, 4 (33.3%) were phenotypically confirmed as ESBL producers (Supporting Information S2: Table S2; Supporting Information S1: Figure S3). Of these four ESBL‐positive isolates, two harbored both *bla*_*CTX‐M*_ and *bla*_*TEM‐1*_ genes, while the remaining two did not carry any of the three tested ESBL‐associated genes (*bla*_*TEM‐1*_, *bla*_*SHV*_, and *bla*_*CTX‐M*_) (Supporting Information S2: Table S2; Supporting Information S1: Figure S4). Overall, seven isolates carried at least one of the three ESBL‐associated genes. Among them, *bla*_*TEM‐1*_ was the most prevalent, detected in six isolates (chi‐square test, *p* > 0.05) (Figure 5A,B; Supporting Information S1: Figure S4; and Supporting Information S2: Table S2). The other two genes, *bla*_*SHV*_ and *bla*_*CTX‐M*_, were each detected in two isolates (Figure 5B; Supporting Information S1: Figure S4; and Supporting Information S2: Table S2). The observed gene combination patterns included *bla*_*TEM‐1*_/*bla*_*SHV*_ (*n* = 1; 8.3%) and *bla*_*TEM‐1*_/*bla*_*CTX‐M*_ (*n* = 2; 16.7%) (Figure 5B). Bivariate analysis was performed to assess potential correlations among the ESBL‐associated genes. Although no statistically significant correlation was found, a moderate association was observed between *bla*_*TEM‐1*_ and *bla*_*CTX‐M*_ (Figure 4B). Furthermore, among the seven isolates harboring at least one of the three ESBL‐associated genes, co‐resistance was most frequently observed against several non‐β‐lactam antibiotics, including ciprofloxacin (*n* = 3), neomycin (*n* = 3), trimethoprim/sulfamethoxazole (*n* = 3), and azithromycin (*n* = 3) (Supporting Information S2: Table S2).

### 3.6 Logistic Regression Analysis

Binary logistic regression was performed to evaluate the association between the presence of ESBL‐associated genes (independent variables) and antimicrobial susceptibility profiles to 13 antimicrobial agents, as well as the presence of *E. coli* pathotypes. The analysis did not reveal any statistically significant associations (*p* > 0.05 for all comparisons) between the presence of any ESBL‐associated genes and resistance to any of the tested antimicrobials or *E. coli* pathotypes (Supporting Information S2: Tables S3 and S4). These findings suggest that the presence of *bla*_*TEM‐1*_, *bla*_*SHV*_, or *bla*_*CTX‐M*_ did not significantly influence the resistance profiles of the tested *E. coli* isolates or the presence of diarrheagenic pathotypes.

## 4 Discussion

The study provides insight into the possibility of the presence of DEC on FTS in public areas. Very little is known about the contamination of FTS in Bangladesh. Furthermore, data highlighting the magnitude of the pathogen burden of these surfaces in different public areas is limited. This study was the first approach to identify DEC‐ and ESBL‐producing *E. coli* from FTS in selected public areas of Bangladesh. Although a study in Bangladesh demonstrated DEC from the household surfaces (Hossain et al. 2021), earlier studies (Chowdhury et al. 2016; Acharjee et al. 2019; Rahman et al. 2019) did not reflect the occurrence of DEC and ESBL on FTS in public areas in Bangladesh. However, infectious diseases transmitted by FTS may be regarded as a major public health concern. Assessment of the microbial quality of these surfaces in household and communal settings is imperative to raise public awareness as well as provide evidence for taking proper preventive interventions.

In our study, DEC was recovered from only two isolates (*n* = 2; 1.9%) from the FTS samples examined in this study. Although DEC was not reported from FTS in public areas, the occurrence of DEC in household environments and clinical patients has been reported with higher frequencies from Bangladesh compared to the present study (Parvej et al. 2020; Rahman et al. 2020; Hossain et al. 2021). In contrast, Moniruzzaman et al. (2023) isolated 117 ESBL‐producing *E. coli* from hospital surfaces and clinical samples, none of which harbored diarrheagenic virulence factors (Moniruzzaman et al. 2023). This relatively low prevalence of DEC identified in the present study contrasts with findings from a recent study conducted in the kitchen and toilet areas of rural households in South Africa, where DEC was detected in 35 out of 105 (33.3%) surface samples (Rakhalaru et al. 2023). The differences in detection rates may be attributed to variations in sampling locations, surface types, methodologies, hygiene practices, or differences in the prevalence patterns of virulence genes in the tested environments. In this study, we have documented the presence of EIEC and DAEC in the FTS. The occurrence of EIEC was also reported earlier from household surfaces in Bangladesh (Hossain et al. 2021), which corresponded to the current findings. However, the occurrence of DAEC from FTS is reported for the first time in our study, to the best of our knowledge. These results were inferred based on a limited screening with 11 virulence determinants in DEC; however, many other potential virulence determinants (*afaB*, *virB*, *astA*, *ial*, and *cdt*) were not studied. Thus, the possibility of the occurrence of other DEC pathotypes could not be excluded. Moreover, previous studies suggested humans as reservoirs of EIEC, EAEC, and DAEC (Parvej et al. 2020). Therefore, we believe that FTS might harbor these three DEC pathotypes, as they are frequently touched by humans. Thus, further studies with a larger sample size along with a wide range of virulence determinants are necessary to ascertain the actual scenario of DEC occurrence on FTS in Bangladesh.

*E. coli* isolated in this study were distributed to all four phylogenetic groups described earlier, with the abundance of A (*n* = 5; 41.6%) and B2 (*n* = 4; 33.3%) comprising commensal and pathogenic groups of *E. coli*, respectively (Halaji et al. 2022). Reports from earlier studies summarized that *E. coli* from the A and B1 phylogenetic groups predominated in animal gut flora, while isolates from the B2 were the most abundant in humans (Aguirre‐Sánchez et al. 2022; Bag et al. 2021; Halaji et al. 2022). B2 *E. coli* primarily belonged to extraintestinal *E. coli* (ExPEC), predominantly reported from urinary tract infections (Halaji et al. 2022). The higher prevalence of B2 *E. coli*, as evident in this study, indicates a possible threat to human health from the acquisition and dissemination of ExPEC through FTS.

AMR is essential for understanding pathogenesis and selecting appropriate antimicrobials to abate *E. coli*‐associated infections. Ten out of 12 isolates (83.3%) in this study were MDR, with the highest resistance being to ampicillin (*n* = 12; 100%), followed by cefotaxime (*n* = 8; 66.7%), trimethoprim/sulfamethoxazole (*n* = 6; 50%), neomycin (*n* = 5; 41.7%), nitrofurantoin (*n* = 5; 41.7%), ciprofloxacin (*n* = 5; 41.7%), and ceftazidime (*n* = 3; 25%). The higher resistance to ampicillin, trimethoprim/sulfamethoxazole, cefotaxime, and ciprofloxacin from this study coincided with the findings described earlier (Talukdar et al. 2013; Zhou et al. 2018; Parvin et al. 2020; Bag et al. 2021). The prolonged and irrational use of these antibiotics in the human and livestock populations of Bangladesh might be the cause of this high resistance. However, these studies demonstrated higher tetracycline resistance, which differed from the present study. In the current investigation, neither imipenem nor gentamicin resistance was observed, while chloramphenicol (*n* = 1; 8.3%) had the lowest resistance levels, which is compatible with other reported findings from Bangladesh (Talukdar et al. 2013; Rashid et al. 2015). Surprisingly, the present study demonstrated 41.7% (*n* = 5) nitrofurantoin resistance in *E. coli*, which is comparatively higher than the reports published earlier in Bangladesh (Haque et al. 2015; Hossain et al. 2020). Since nitrofurantoin is highly concentrated in urine, it appears to be the first‐line targeted antimicrobial for the treatment of UTI patients (Mahdizade Ari et al. 2023). As a result of its widespread application to UTI patients in Bangladesh (Hossain et al. 2020), resistance is gradually growing, which could alarmingly lower therapeutic efficiency. Therefore, more controlled approaches to antibiotic prescriptions in different sectors are desirable.

The observed strong positive correlations between phenotypic resistances—such as between chloramphenicol and gentamicin, and between cefotaxime and multiple other antibiotics—may indicate co‐resistance due to the presence of linked resistance genes on mobile genetic elements. These findings suggest potential co‐selection and the presence of MDR strains circulating in the environment. Furthermore, the median MARI of the 12 *E. coli* isolates was 0.34 (range: 0.08–0.62), with 10 (*n* = 10; 83.3%) isolates exhibiting MARI > 0.2. The high MARI values observed align with findings from Beshiru et al. (2022) (0.94) and Fallah et al. (2021) (0.75). A MAR index above 0.2 is considered a critical threshold, suggesting that these bacteria originate from environments with high antibiotic exposure, such as hospitals, livestock farms, or areas with frequent antibiotic misuse (Siri et al. 2024). The presence of such high MAR values suggests that a significant proportion of the isolates originate from high‐risk environments where multiple antibiotics are used. FTS in hospitals, public restrooms, and other public areas serve as hotspots for bacterial contamination due to constant human contact. These surfaces can act as fomites, facilitating the transmission of MDR *E. coli* among individuals. Hospitals, in particular, are considered breeding grounds for AMR, with potential dissemination to other public areas due to improper hand hygiene. The findings of high MAR values in our study reinforce the need for proper hygiene practices and effective disinfection protocols in public spaces. Additionally, the widespread resistance observed in our isolates highlights the necessity for stricter antibiotic stewardship programs, surveillance of resistance trends, and policies to control antibiotic misuse in both clinical and agricultural settings.

Although several previous studies have reported ESBL‐producing *E. coli* from Bangladesh (Rashid et al. 2015; Mahmud et al. 2020; Islam et al. 2022), until now, their relationship with FTS has yet to be investigated. Out of 12, only 4 *E. coli* isolates (*n* = 4; 33.3%) were phenotypically identified as ESBL‐producing, which is higher than the study conducted by Mahmud et al. (2020) but lower than the occurrence reported by Islam et al. (2022). The present study demonstrated *bla*_*TEM‐1*_ (*n* = 6; 50%) as predominant over the other ESBL‐associated genes, which is consistent with the studies conducted in Bangladesh (Parvin et al. 2020; Islam et al. 2022) and Nigeria (Ugbo et al. 2020). However, Moniruzzaman et al. (2023) reported the highest prevalence of the *bla*_*CTX‐M*_ gene (68.4%) out of 117 ESBL *E. coli* isolates from hospital environments and clinical patients from Bangladesh. Moreover, many studies conducted at home (Talukdar et al. 2013; Rashid et al. 2015) and abroad (Dhawde et al. 2018; Zhou et al. 2018) recently demonstrated the high prevalence of the *bla*_*CTX‐M*_ gene, which is regarded as the most predominant ESBL‐associated gene isolated from humans (Zhou et al. 2018). Literature suggests that the presence of the *bla*_*CTX‐M*_ gene is a strong indicator of ESBL production. Studies have shown that detecting *bla*_*CTX‐M*_ genes in bacterial isolates is highly correlated with ESBL activity (Abe et al. 2019; Legese et al. 2022). In contrast, *bla*_*TEM*_ and *bla*_*SHV*_ genes can encode both ESBL and non‐ESBL enzymes. Therefore, the presence of these genes alone does not confirm ESBL production without further characterization (Legese et al. 2022). Consistent with previous reports, both isolates carrying the *bla*_*CTX‐M*_ gene in this study exhibited phenotypic ESBL production. However, five isolates carrying either the *bla*_*TEM‐1*_ and *bla*_*SHV*_ gene or a combination of both were identified as non‐ESBL producers by the DDST. Interestingly, two of the phenotypically ESBL‐positive *E. coli* isolates did not harbor any of the three tested ESBL‐associated genes. A similar observation was reported by Chaudhary et al. (2023), suggesting the possibility of false‐positive results by phenotypic methods or the presence of other ESBL gene families such as SFO, BES, BEL, TLA, GES, PER, VEB, and OXA, which were not targeted in the current study. Alternatively, resistance may be mediated by nonenzymatic mechanisms, such as structural modifications in penicillin‐binding proteins, which reduce β‐lactam binding affinity and confer resistance independently of classical ESBL enzymes. This highlights a limitation in the gene panel used and underscores the need for more comprehensive molecular screening in future investigations to better characterize the genetic basis of ESBL production.

Although the B1 phylogenetic group of *E. coli* is primarily associated with commensal strains shed by animals, one ESBL‐producing isolate in this study belonged to this group and carried the diarrheagenic EIEC virulence, as reported by Hazen et al. (2016). This strain also harbored both the *bla*_*CTX‐M*_ and *bla*_*TEM‐1*_ genes, likely acquired through horizontal gene transfer from a pathogenic strain. The presence of resistance or virulence genes in commensal strains underscores the threat of horizontal gene transfer, highlighting their potential pathogenicity and limiting therapeutic options.

It is worth noting that a nonsignificant moderate correlation was observed between *bla*_*TEM‐1*_ and *bla*_*CTX‐M*_ in the present study, indicating the abundance of these two ESBL genes over other ESBL genes, which is in line with previous reports (Dhawde et al. 2018). However, the present study could not demonstrate the subtypes of *bla*_*CTX‐M*_, which would be more informative. Interestingly, four *E. coli* isolates were not positive for any of the examined ESBL‐associated genes despite their phenotypic resistance to third‐generation cephalosporins (cefotaxime and ceftazidime). These isolates might encode distinct ESBL types (PER, GES, VEB, TLA, SFO, BEL, BES, and OXA) (Bubpamala et al. 2018) from those investigated in this study.

Since ESBL genes are frequently co‐located with other resistance determinants on plasmids, their presence not only confers resistance to extended‐spectrum cephalosporins but also facilitates resistance to unrelated antibiotic classes through co‐selection. Consequently, the present study also observed co‐resistance of ESBL‐associated genes to ciprofloxacin, neomycin, trimethoprim/sulfamethoxazole, and azithromycin. This finding aligns with the recent report by Soni et al. (2024), which documented the coexistence of ESBL genes with resistance to trimethoprim, ciprofloxacin, azithromycin, tetracycline, vancomycin, gentamicin, and colistin in *E. coli* isolates from wastewater samples in India. This co‐resistance pattern suggests the potential linkage of ESBL genes with other AMR determinants, possibly mediated through mobile genetic elements such as plasmids or integrons. Surprisingly, this study did not find any statistically significant associations between ESBL genes and resistance to the tested antimicrobials, suggesting that other resistance mechanisms may be contributing to the observed resistance patterns. Additionally, the small sample size could have influenced the inability to detect significant associations, as it is well established that logistic regression models require an adequate number of events per variable (at least 10 positive events per variable) to produce reliable estimates (Van Smeden et al. 2019). Further studies with larger sample sizes and additional genetic markers may be required to elucidate these associations.

This study systematically sampled and measured the magnitude of bacterial contamination on frequently touched environmental surfaces in public areas, which ranged from 6.4 to 8.56 Log10 CFU/cm^2^. The highest average bacterial load was identified from the keyboard of ATM surfaces (8.56 Log10 CFU/cm^2^), which was comparatively more elevated than the reported bacterial contamination from hospital surfaces (Claro et al. 2015; Odoyo et al. 2021). Surprisingly, this surface did not harbor *E. coli*, indicating the possibility of harboring other health‐hazardous pathogens like MRSA, VRE, and so forth, as pointed out earlier (Acharjee et al. 2019). However, the possibility of the presence of other pathogenic bacteria needs to be further investigated.

The findings of this study highlight a critical public health concern due to the dispersal of DEC and ESBL‐producing strains on commonly touched surfaces in public areas. Results like these emphasize the growing necessity of improved sanitation in public spaces, especially in social hotspots like hospitals, shopping malls, and public restrooms. Strengthening routine disinfection measures for high‐touch surfaces and promoting proper hand hygiene practices could help mitigate the risk of environmental dissemination and pathogen transmission. Government agencies should enhance efforts to improve hygienic conditions in public areas. Additionally, mass media, print media, and public health departments can play an active role in educating the public about personal hygiene and the consequences of neglecting hand hygiene on public health. Furthermore, the identification of virulence‐associated *E. coli* strains on public surfaces highlights the need for more stringent environmental surveillance programs. Policymakers should consider implementing regular microbial monitoring of FTS to assess contamination levels and evaluate the effectiveness of sanitation efforts. Given the role of antimicrobial‐resistant bacteria, stringent regulations on antibiotic use in both clinical and environmental settings should be reinforced to limit the emergence and dissemination of resistant strains.

As far as is known, this is the first approach in Bangladesh to assess the diarrheagenic potentiality of *E. coli* from publicly shared common touch surfaces; however, the study acknowledges several limitations, particularly regarding the sample size, which resulted in the recovery of a relatively small number of *E. coli* isolates (*n* = 12) from 105 surface samples, with only two isolates identified as diarrheagenic. This low recovery rate inevitably reduces the statistical power of the analysis and limits our ability to detect meaningful associations and generalize the findings. However, the study was designed as an exploratory investigation to assess the presence and types of *E. coli* across a broad range of environmental surface samples, where pathogen prevalence is inherently low and variable. A formal power analysis was not conducted due to the lack of baseline prevalence data in similar settings. Future studies with larger sample sizes or more targeted sampling strategies may help strengthen statistical inference and provide a more robust understanding of contamination patterns. Additionally, the present study could not afford control strains of each DEC pathotype and ESBL strain, demonstrate the phenotypic evidence of the virulence factors, MIC of widely used antibiotics in humans (imipenem, β‐lactam antibiotics, and ciprofloxacin), and phylogenetic relationship among the DEC strains due to insufficient laboratory facilities and funding, which would be more informative. The present study only focused on the most prevalent ESBL gene families (*bla*_*TEM‐1*_, *bla*_*SHV*_, and *bla*_*CTX‐M*_). However, we acknowledge that other important ESBL genes, such as *bla*_*PER*_, *bla*_*VEB*_, *bla*_*GES*_, and *bla*_*OXA*_, also play significant roles in resistance dissemination. Future studies should expand the molecular screening to include a more comprehensive panel of ESBL and other β‐lactamase genes to provide a broader understanding of resistance mechanisms. Moreover, phenotypic evidence of ESBL was not evaluated with other reported methods (Mahmud et al. 2020). The present study could not demonstrate any strong evidence between EIEC and *Shigella* spp., as both have been found to possess the *ipaH* gene. Hence, further detailed studies with a larger sample size, an extensive coverage area, a diverse microbial community, and in‐depth genetic analysis of the DEC pathotypes and AMR with diverse ESBL types, AmpC β‐lactamase, quinolone, and carbapenemase genes are needed to ascertain the actual implications of publicly shared common touch surfaces on human health.

## 5 Conclusions

This study reveals significant microbial contamination on frequently touched public surfaces, with *E. coli* detected in 11.43% (*n* = 12) of samples. Two isolates carried diarrheagenic virulence genes (*ipaH* and *daaD*), while 83.3% were MDR and 33.3% exhibited ESBL production. These findings highlight the potential public health risks posed by environmental reservoirs of antimicrobial‐resistant bacteria. They underscore the urgent need for enhanced hygiene practices to reduce pathogen burden in public spaces. Routine environmental surveillance is also essential to better understand the role of commonly touched surfaces in the transmission of community‐acquired infections. Furthermore, the results reinforce the importance of prudent antibiotic use across all sectors, particularly in clinical settings, with a strong emphasis on antimicrobial susceptibility testing. A coordinated global One Health approach is critical to combating the growing threat of AMR and safeguarding public health.

## Author Contributions

**Mohammad Arif:** conceptualization, methodology, investigation, formal analysis, writing – initial draft, writing – review and editing. **Asma Ul Hosna:** investigation, methodology, writing – initial draft. **Ishrat Jahan:** investigation, methodology. **Md. Ashiquen Nobi:** investigation, methodology. **Most. Shumi Akhter Shathi:** writing – initial draft, writing – review and editing. **MD. Nazmul Hasan:** formal analysis, conceptualization, writing – review and editing. **Jayedul Hassan:** supervision, conceptualization, methodology, formal analysis, writing – review and editing. **S. M. Lutful Kabir:** supervision, conceptualization, methodology, formal analysis, writing – review and editing, funding acquisition.

## Ethics Statement

As no animal or human subject was employed, no ethical approval was needed for this study. However, verbal consent from the respective authority was obtained during the collection of samples.

## Conflicts of Interest

The authors declare no conflicts of interest.

## Supporting information


**Figure S1:** Representative photograph of diarrheagenic *E. coli* isolated in this study. A) *ipaH* gene amplification of the *E. coli* by PCR, indicating the Enteroinvasive pathotype. Lane 1: *E. coli* isolate of this study. B) *daaD* gene amplification of the *E. coli* by PCR, indicating the Diffusely‐adherent *E. coli* pathotype. Lane 1: *E. coli* isolate of this study. **Figure S2:** Representative photograph of phylogenetic grouping of all *E. coli* isolated in this study by PCR. A) Phylogenetic grouping of *E. coli* by PCR targeting the *chuA* gene showing positive band at 279 bp. Lanes 1‐4: *E. coli* isolates of this study. B) Phylogenetic grouping of *E. coli* by PCR targeting the *yjaA* gene showing positive band at 211 bp. Lanes 2‐4: *E. coli* isolates of this study. C) Phylogenetic grouping of *E. coli* by PCR targeting the DNA fragment TspE4.c2 showing positive band at 152 bp. Lanes 1‐2: *E. coli* isolates of this study; Lane 3: *E. coli* strain ATCC25922 (control). **Figure S3:** Representative photograph of double disk synergy test to identify the ESBL‐producing *E. coli*. **Figure S4:** Representative photograph of ESBL gene amplification of *E. coli* isolated in this study. A) *bla*TEM‐1 gene amplification by PCR showing positive band at 643 bp. Lanes 1‐6: *E. coli* isolates of this study. B) *blaSHV* gene amplification by PCR showing positive band at 714 bp. Lanes 1‐2: *E. coli* isolates of this study. C) *bla*CTX‐M gene amplification by PCR showing positive band at 766 bp. Lanes 1‐2: *E. coli* isolates of this study.

**Table S1:** List of primers used in this study. **Table S2:** Virulence properties and antimicrobial resistance of all the *E. coli* isolated in this study. **Table S3:** Binary logistic regression analysis of the association between ESBL‐associated genes and antimicrobial resistance profiles of 12 *E. coli* isolates in this study. **Table S4:** Binary logistic regression analysis of the association between ESBL‐associated genes and status of *E. coli* pathotypes (presence or absence), identified in this study.

## Data Availability

The data sets of the current study are available from the corresponding author on reasonable request.
