# Supplementary material for: Diarrheagenic and ESBL Potential of Escherichia coli From Publicly Shared Common Touch Surfaces
Source: Microbiologyopen. 2025 Nov 11;14(6):e70125. doi: 10.1002/mbo3.70125 (PMC12606042; doi:10.1002/mbo3.70125)
Supplement: Supplementary file 1 — Figure S1: Representative photograph of diarrheagenic E. coli isolated in this study. A) ipaH gene amplification of the E. coli by PCR, indicating the Enteroinvasive pathotype. Lane 1: E. coli isolate of this study. B) daaD gene amplification of the E. coli by PCR, indicating the Diffusely‐adherent E. coli pathotype. Lane 1: E. coli isolate of this study. Figure S2: Representative photograph of phylogenetic grouping of all E. coli isolated in this study by PCR. A) Phylogenetic grouping of E. coli by PCR targeting the chuA gene showing positive band at 279 bp. Lanes 1‐4: E. coli isolates of this study. B) Phylogenetic grouping of E. coli by PCR targeting the yjaA gene showing positive band at 211 bp. Lanes 2‐4: E. coli isolates of this study. C) Phylogenetic grouping of E. coli by PCR targeting the DNA fragment TspE4.c2 showing positive band at 152 bp. Lanes 1‐2: E. coli isolates of this study; Lane 3: E. coli strain ATCC25922 (control). Figure S3: Representative photograph of double disk synergy test to identify the ESBL‐producing E. coli. Figure S4: Representative photograph of ESBL gene amplification of E. coli isolated in this study. A) blaTEM‐1 gene amplification by PCR showing positive band at 643 bp. Lanes 1‐6: E. coli isolates of this study. B) blaSHV gene amplification by PCR showing positive band at 714 bp. Lanes 1‐2: E. coli isolates of this study. C) blaCTX‐M gene amplification by PCR showing positive band at 766 bp. Lanes 1‐2: E. coli isolates of this study. [file MBO3-14-e70125-s001.docx]

**Supplementary Figures**

**Diarrheagenic and ESBL potential of *Escherichia coli* from publicly shared common touch surfaces**

Mohammad Arif^1,^*^ϯ^*, Asma Ul Hosna^1,^*^ϯ^*, Ishrat Jahan^1^, Md. Ashiquen Nobi^1^, Most. Shumi Akhter Shathi^2^, MD Nazmul Hasan^3^, Jayedul Hassan^1^, S. M. Lutful Kabir^1^

^1^Department of Microbiology and Hygiene, Bangladesh Agricultural University, Mymensingh-2202, Bangladesh

^2^Department of Pharmacology, Bangladesh Agricultural University, Mymensingh-2202, Bangladesh

^3^Department of Medicine, Bangladesh Agricultural Univeristy, Mymensingh-2202, Bangladesh

**^ϯ^Both authors contributed equally**

**Correspondence:** S. M. Lutful Kabir

Email address: lkabir79@bau.edu.bd

ORCID ID: https://orcid.org/0000-0003-3684-3387

Postal address: S. M. Lutful Kabir, Department of Microbiology and Hygiene, Bangladesh Agricultural University, Mymensingh-2202, Bangladesh; Mobile: +8801754987218; Tel.: +88-091-67401-6/Ext. 63218; Fax: +88-091-61510


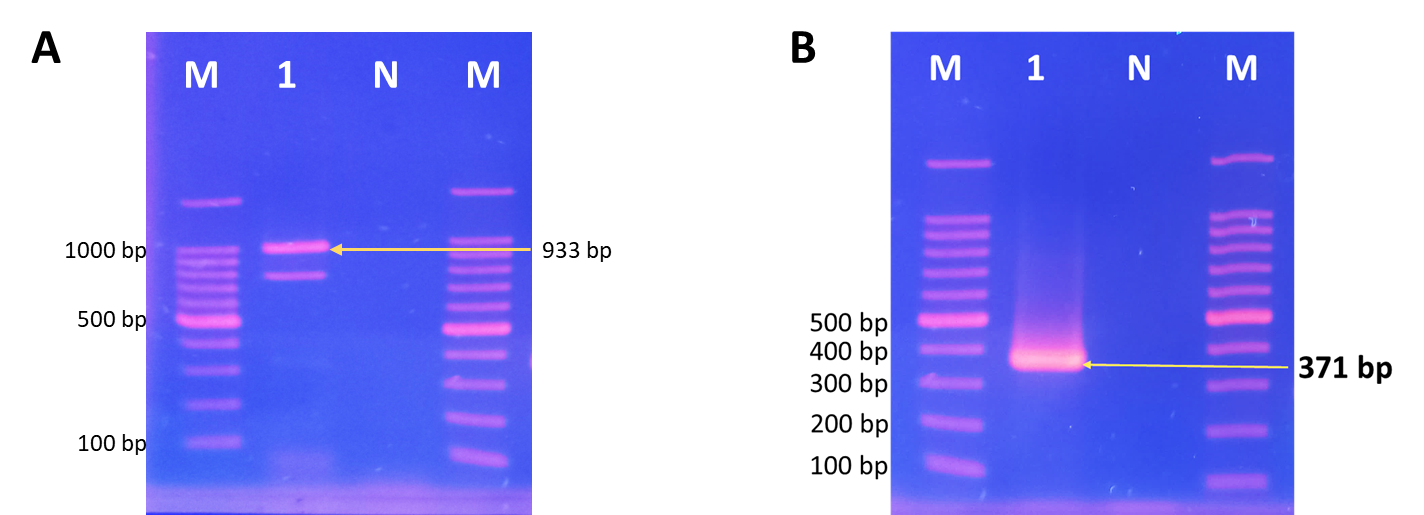


**Figure S1:** Representative photograph of diarrheagenic *E. coli* isolated in this study. A) *ipaH* gene amplification of the *E. coli* by PCR, indicating the Enteroinvasive pathotype. Lane 1: *E. coli* isolate of this study. B) *daaD* gene amplification of the *E. coli* by PCR, indicating the Diffusely-adherent *E. coli* pathotype. Lane 1: *E. coli* isolate of this study. In all cases, Lane N: *E. coli* strain ATCC25922 (control); Lane M: 100 bp DNA ladder, Promega. The electrophoresis was carried out using 1.5% agarose gel (Promega) at 100 volts for 25 minutes in 1x TAE buffer.


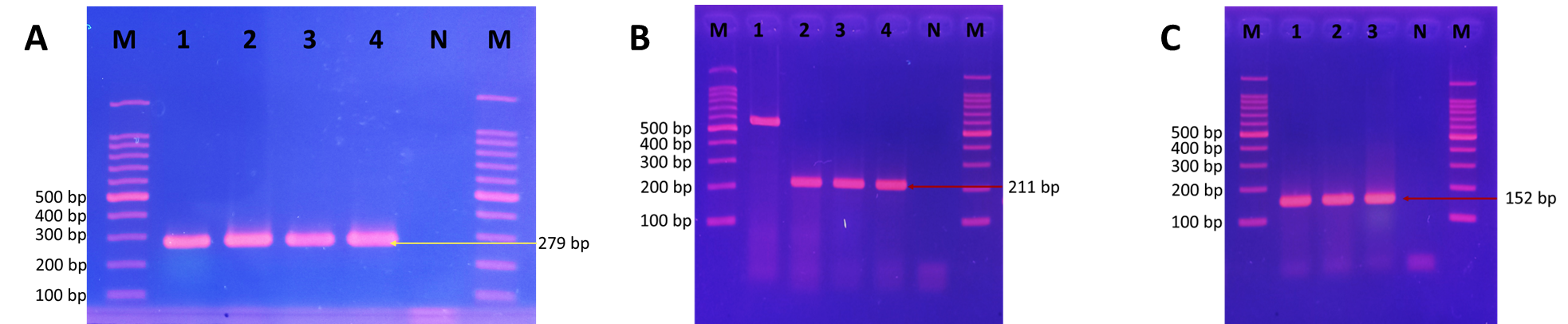


**Figure S2:** Representative photograph of phylogenetic grouping of all *E. coli* isolated in this study by PCR. A) Phylogenetic grouping of *E. coli* by PCR targeting the *chuA* gene showing positive band at 279 bp. Lanes 1-4: *E. coli* isolates of this study. B) Phylogenetic grouping of *E. coli* by PCR targeting the *yjaA* gene showing positive band at 211 bp. Lanes 2-4: *E. coli* isolates of this study. C) Phylogenetic grouping of *E. coli* by PCR targeting the DNA fragment TspE4.c2 showing positive band at 152 bp. Lanes 1-2: *E. coli* isolates of this study; Lane 3: *E. coli* strain ATCC25922 (control). In all cases, Lane N: Negative control without DNA samples; Lane M: 100 bp DNA ladder, Promega. The electrophoresis was carried out using 1.5% agarose gel (Promega) at 100 volts for 25 minutes in 1x TAE buffer.

**Figure S3:** Representative photograph of double disk synergy test to identify the ESBL-producing *E. coli*. The test was performed using ceftazidime (CAZ), cefotaxime (CTX), and amoxicillin-clavulanic acid (AMC) disks. While the CAZ disk did not show an increased zone of inhibition, the CTX disk exhibited an expansion of approximately 13 mm toward the AMC disk, indicating ESBL production.


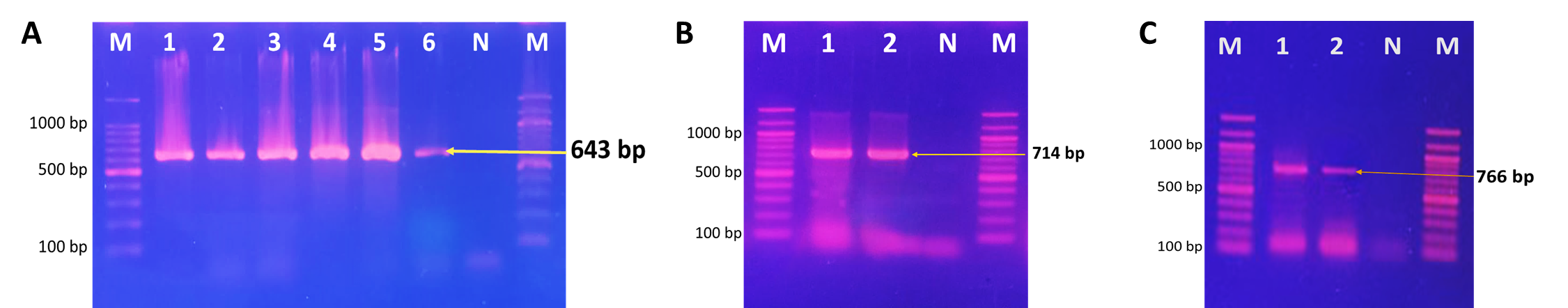


**Figure S4:** Representative photograph of ESBL gene amplification of *E. coli* isolated in this study. A) *bla*_TEM-1_ gene amplification by PCR showing positive band at 643 bp. Lanes 1-6: *E. coli* isolates of this study. B) *bla_SHV_* gene amplification by PCR showing positive band at 714 bp. Lanes 1-2: *E. coli* isolates of this study. C) *bla*_CTX-M_ gene amplification by PCR showing positive band at 766 bp. Lanes 1-2: *E. coli* isolates of this study. In all cases, Lane N: *E. coli* strain ATCC25922 (control); Lane M: 100 bp DNA ladder, Promega. The electrophoresis was carried out using 1.5% agarose gel (Promega) at 100 volts for 25 minutes in 1x TAE buffer.
