# Supplementary material for: Diarrheagenic and ESBL Potential of Escherichia coli From Publicly Shared Common Touch Surfaces
Source: Microbiologyopen. 2025 Nov 11;14(6):e70125. doi: 10.1002/mbo3.70125 (PMC12606042; doi:10.1002/mbo3.70125)
Supplement: Supplementary file 2 — Table S1: List of primers used in this study. Table S2: Virulence properties and antimicrobial resistance of all the E. coli isolated in this study. Table S3: Binary logistic regression analysis of the association between ESBL‐associated genes and antimicrobial resistance profiles of 12 E. coli isolates in this study. Table S4: Binary logistic regression analysis of the association between ESBL‐associated genes and status of E. coli pathotypes (presence or absence), identified in this study. [file MBO3-14-e70125-s002.doc]

**Supplementary Tables**

**Diarrheagenic and ESBL potential of *Escherichia coli* from publicly shared common touch surfaces**

Mohammad Arif^1^,*^ϯ^*, Asma Ul Hosna^1^,*^ϯ^*, Ishrat Jahan^1^, Md. Ashiquen Nobi^1^, Most. Shumi Akhter Shathi^2^, MD Nazmul Hasan^3^, Jayedul Hassan^1^, S. M. Lutful Kabir^1^

^1^Department of Microbiology and Hygiene, Bangladesh Agricultural University, Mymensingh-2202, Bangladesh

^2^Department of Pharmacology, Bangladesh Agricultural University, Mymensingh-2202, Bangladesh

^3^Department of Medicine, Bangladesh Agricultural Univeristy, Mymensingh-2202, Bangladesh

**^ϯ^Both authors contributed equally**

**Correspondence:** S. M. Lutful Kabir

Email address: lkabir79@bau.edu.bd

ORCID ID: https://orcid.org/0000-0003-3684-3387

Postal address: S. M. Lutful Kabir, Department of Microbiology and Hygiene, Bangladesh Agricultural University, Mymensingh-2202, Bangladesh; Mobile: +8801754987218; Tel.: +88-091-67401-6/Ext. 63218; Fax: +88-091-61510

**Table S1:** **List of primers used in this study.**

| **Purpose** | **Target genes/DNA fragment** | **Forward primer** | **Reverse primer** | **Annealing temperature (^o^C)** | **Amplicon size** | **Reference** |
| --- | --- | --- | --- | --- | --- | --- |
| **Identification of *E. coli*** | | | | |  | |
|  | *malB* | GACCTCGGTTTAGTTCACAGA | CACACGCTGACGCTGACCA | 52 | 585bp | Wang et al. 1996 |
| **Phylogenetic grouping of *E. coli*** | | | | |  | |
|  | *chuA* | GACGAACCAACGGTCAGGAT | TGCCGCCAGTACC AAAGACA | 55 | 279bp | Clermont et al. 2000 |
|  | *yjaA* | TGAAGTGTCAGGAGACGCTG | ATGGAGAATGCGTTCCTCAAC |  | 211bp |  |
|  | TspE4C2 | GAGTAATGTCGGGGCATTCA | CGCGCCAACAAAGTATTACG |  | 152bp |  |
| **Virulence genes of *E. coli*** | | | | |  | |
| *E. coli* O157 | *rfbE* | CGGACATCCATGTGATATGG | TTGCCTATGTACAGCTAATCC | 60 | 259bp | Paton and Paton 1993 |
| STEC | *stx_1_* | CACAATCAGGCGTCGCCAGCGCACTTGCT | TGTTGCAGGGATCAGTCGTACGGGGATGC | 58 | 606bp | Talukdar et al. 2013 |
|  | *stx_2_* | CCACATCGGTGTCTGTTATTAACCACACC | GCAGAACTGCTCTGGATGCATCTCTGGTC |  | 372bp | Talukdar et al. 2013 |
| EPEC | *eaeA* | TCAATGCAGTTCCGTTATCAGTT | GTAAAGTCCGTTACCCCAACCTG | 61 | 482bp | Fallah et al. 2021 |
|  | *bfpA* | GGAAGTCAAATTCATGGGGGTAT | GGAATCAGACGCAGACTGGTAGT |  | 300bp |  |
| EIEC | *ipaH* | CTCGGCACGTTTTAATAGTCTGG | GTGGAGAGCTGAAGTTTCTCTGC | 60 | 933bp | Rúgeles et al. 2010 |
|  | *virF* | AGCTCAGGCAATGAAACTTTGAC | TGGGCTTGATATTCCGATAAGTC |  | 618bp | Canizalez-Roman et al. 2013 |
| ETEC | ST | AGGAACGTACATCATTGCCC | CAAAGCATGCTCCAGCACTA | 53 | 521bp | Sobhy et al. 2020 |
|  | LT | GGCGTTACTATCCTCTCTAT | TGGTCTCGGTCAGATATGT | 56 | 272bp | Girard et al. 2020 |
| EAEC | *agg*R | GTATACACAAAAGAAGGAAGC | ACAGAATCGTCAGCATCAGC | 59 | 254bp | Fallah et al. 2021 |
|  | *pCVD432* | CTGGCGAAAGACTGTATCAT | AATGTATAGAAATCCGCTGTT | 55 | 630bp |  |
| DAEC | *daaD* | TGAACGGGAGTATAAGGAAGATG | GTCCGCCATCACATCAAAA | 56 | 371bp | Fallah et al. 2021 |
| **ESBL producing genes/Antimicrobial resistance genes** | | | | |  | |
|  | *bla_TEM-1_* | CAGCGGTAAGATCCT TGAGA | ACT CCC CGT CGT GTA GAT AA | 56 | 643bp | Chen et al. 2004 |
|  | *bla_SHV_* | GGCCGCGTAGGCATGATAGA | CCCGGCGATTTGCTG ATTTC |  | 714bp |  |
|  | *bla_CTX-M_* | AACCGTCACGCTGTTGTTAG | TTGAGGCTGGGTGAAGTAAG | 50 | 766bp |  |

STEC: Shiga toxin producing *E. coli*, EPEC: Enteropathogenic *E. coli*, EIEC: Enteroinvasive *E. coli*, ETEC: Enterotoxigenic *E. coli*, EAEC: Enteroaggregative *E. coli*, DAEC: Diffusely adherent *E. coli*, ID: Initial denaturation, D: Denaturation, A: Annealing, E: Extension, FE: Final extension, bp: Base pair

**Table S2: Virulence properties and antimicrobial resistance of all the *E. coli* isolated in this study.**

| **Sl. No.** | **Sample code** | **Virulence genes** | | | | | | | | | | | | ***E. coli* pathotype** | **Antibiotic resistance pattern** | **No. of antibiotics (Classes)** | **No. (%) of MDR isolates**  **(n = 12)** | **Overall no. (%) of MDR isolates** | **MARI** | **Antimicrobial resistance genes** | | | **ESBL Producing *E. coli*** | **Phylogenetic group** |
| --- | --- | --- | --- | --- | --- | --- | --- | --- | --- | --- | --- | --- | --- | --- | --- | --- | --- | --- | --- | --- | --- | --- | --- | --- |
|  |  | ***rfbE* O157** | ***stx1*** | ***stx2*** | ***eaeA*** | ***bfpA*** | ***ipaH*** | ***virF*** | **ST** | **LT** | ***aggR*** | **pCVD432** | ***daaD*** |  |  |  |  |  |  | ***bla*_TEM-1_** | ***bla*_SHV_** | ***bla*_CTX-M_** |  |  |
| 1 | PTG02 | - | - | - | - | - | - | - | - | - | - | - | - | - | AMP-CTX-SXT-N-F-CAZ | 6 (5) | 1 (8.33) | 10 (83.33) | 0.46 | - | - | - | + | B2 |
| 2 | EB06 | - | - | - | - | - | - | - | - | - | - | - | + | DAEC | AMP-CTX-SXT-CIP-AZM-CAZ | 6 (5) | 1 (8.33) |  | 0.46 | + | + | - | - | D |
| 3 | TDN14 | - | - | - | - | - | - | - | - | - | - | - | - | - | AMP-SXT-CIP-TE | 4 (4) | 1 (8.33) |  | 0.31 | + | - | - | - | A |
| 4 | TDN15 | - | - | - | - | - | - | - | - | - | - | - | - | - | AMP-CTX-SXT-N-CIP-AZM-TE-C | 8 (8) | 1 (8.33) |  | 0.62 | + | - | - | - | B1 |
| 5 | TFB08 | - | - | - | - | - | - | - | - | - | - | - | - | - | AMP-CTX-CIP-AZM-CAZ-AMC | 6 (4) | 1 (8.33) |  | 0.46 | - | - | - | - | B2 |
| 6 | TTWK02 | - | - | - | - | - | - | - | - | - | - | - | - | - | AMP-SXT-F-AMC | 4 (3) | 1 (8.33) |  | 0.31 | - | + | - | - | A |
| 7 | TTWK03 | - | - | - | - | - | - | - | - | - | - | - | - | - | AMP-CTX-F | 3 (3) | 1 (8.33) |  | 0.23 | + | - | + | + | B2 |
| 8 | TTWK04 | - | - | - | - | - | - | - | - | - | - | - | - | - | AMP-CTX-N-F-CIP | 5 (5) | 1 (8.33) |  | 0.38 | - | - | - | - | A |
| 9 | TTWK05 | - | - | - | - | - | - | - | - | - | - | - | - | - | AMP-CTX-SXT-F-AZM | 5 (5) | 1 (8.33) |  | 0.38 | - | - | - | + | B2 |
| 10 | TTWK09 | - | - | - | - | - | - | - | - | - | - | - | - | - | AMP | 1 (1) | - |  | 0.08 | - | - | - | - | A |
| 11 | TTWK10 | - | - | - | - | - | - | - | - | - | - | - | - | - | AMP-N | 2 (2) | - |  | 0.15 | + | - | - | - | A |
| 12 | TTWK14 | - | - | - | - | - | + | - | - | - | - | - | - | EIEC | AMP-CTX-N-AZM | 4 (4) | 1 (8.33) |  | 0.31 | + | - | + | + | B1 |

MDR: Multidrug Resistance, MARI: Multiple Antibiotic Resistance Index, DAEC: Diffusely adherent *E. coli*, EIEC: Enteroinvasive *E. coli*, AMP: Ampicillin (µg) 10 µg, CTX: Cefotaxime (30 µg), SXT: Trimethoprim/Sulfamethoxazole (25 µg), N: Neomycin (30µg), F: Nitrofurantoin (300 µg), CIP: Ciprofloxacin (5 µg), AZM: Azithromycin (15 µg), CAZ: Ceftazidime (30 µg), AMC Amoxycillin/Clavulanic Acid (30 µg), TE: Tetracycline (30µg), C: Chloramphenicol (30 µg), PTG: Public transport grab rail sample, EB: Elevator button sample, TDN: Toilet door knob sample, TFB: Toilet flush button sample, TTWK: Toilet tap water knob sample

**Table S3. Binary logistic regression analysis of the association between ESBL-associated genes and antimicrobial resistance profiles of 12 *E. coli* isolates in this study.** ESBL-associated genes were considered independent variables, while antimicrobial resistance profiles served as dependent variables. To fit the binary logistic regression model, antimicrobial resistance profiles were categorized into two groups: resistant (including both intermediate and resistant isolates) and susceptible. A p-value < 0.05 was considered statistically significant.

| **Antimicrobial agents** | ***bla_TEM-1_*  (OR, 95% CI, p-value)** | ***bla_SHV_*  (OR, 95% CI, p-value)** | ***bla_CTX-M_* (OR, 95% CI, p-value)** |
| --- | --- | --- | --- |
| Neomycin | - | - | - |
| Azithromycin | 1.94 (0.14–26.35, p = 0.62) | 1.56 (0.07–36.98), p = 0.79) | 1.15 (0.03–36.49, p = 0.95) |
| Ceftazidime | 0.58 (0.03–11.15, p = 0.72) | 3.27 (0.12–86.47, p = 0.48) | 0 (0–, p = 0.99) |
| Tetracycline | NC (0–, p = 0.99) | 0 (0–, p = 0.99) | 0 (0–, p = 0.99) |
| Cefotaxime | 0.13 (0.005–3.25, p = 0.21) | NC (0–, p = 0.99) | NC (0–, p = 0.99) |
| Amoxicillin/ clavulanic acid | 1.33 (0.07–26.62, p = 0.85) | NC (0–, p = 0.99) | 0.5 (0.01–19.56, p = 0.71) |
| Nitrofurantoin | 0.5 (0.02–12.90, p = 0.68) | NC (0–, p = 0.99) | NC (0–, p = 0.99) |
| Ampicillin | - | - | - |
| Ciprofloxacin | 3.26 (0.19–56.18, p = 0.42) | 0.49 (0.02–13.46, p = 0.68) | 0.27 (0.01–11.48, p = 0.50) |
| Cloramphenicol | 2.00 (0.08–51.59, p = 0.68) | 0 (0–, p = 0.99) | 0 (0–, p = 0.99) |
| Trimethoprim/ Sulfamethoxazole | 3.00 (0.15–59.89, p = 0.47) | NC (0–, p = 0.99) | 0 (0–, p = 0.99) |
| Imipenem | 0 (0–, p = 0.99) | 0 (0–, p = 0.99) | 0.68 (0–, p = 1.00) |
| Gentamicin | NC (0–, p = 0.99) | 0 (0–, p = 0.99) | 0 (0–, p = 0.99) |

NC, Not calculable (extremely high odds ratio); OR, Odds ratio; CI, Confidence interval

**Table S4. Binary logistic regression analysis of the association between ESBL-associated genes and status of *E. coli* pathotypes (presence or absence), identified in this study.** ESBL-associated genes were considered independent variables, while status of *E. coli* pathotypes served as dependent variable. A p-value <0.05 was considered statistically significant.

| **Variable** | **OR** | **95% CI** | | **p-value** |
| --- | --- | --- | --- | --- |
|  |  | **lower** | **Upper** |  |
| *bla_TEM-1_* | NC | 0.00 | . | 0.99 |
| *bla_SHV_* | NC | 0.00 | . | 0.99 |
| *bla_CTX-M_* | NC | 0.00 | . | 0.99 |
| Constant | 0.00 |  |  | 0.99 |

NC, Not calculable (extremely high odds ratio); OR, Odds ratio; CI, Confidence interval

**References:**

Canizalez-Roman, A., Gonzalez-Nuñez, E., Vidal, J.E., Flores-Villaseñor, H. and León-Sicairos, N., 2013. Prevalence and antibiotic resistance profiles of diarrheagenic *Escherichia coli* strains isolated from food items in northwestern Mexico. *International Journal of Food Microbiology*, 164(1), pp.36-45.

Chen, S., Zhao, S., White, D.G., Schroeder, C.M., Lu, R., Yang, H., McDermott, P.F., Ayers, S. and Meng, J., 2004. Characterization of multiple-antimicrobial-resistant *Salmonella* serovars isolated from retail meats. *Applied and Environmental Microbiology*, 70(1), pp.1-7.

Clermont, O., Bonacorsi, S. and Bingen, E. (2000) ‘Rapid and simple determination of the *Escherichia coli* phylogenetic group’, *Applied and environmental microbiology*, 66(10), pp. 4555-4558. Available at: https://doi.org/10.1128/AEM.66.10.4555-4558.2000.

Fallah, N., Ghaemi, M., Ghazvini, K., Rad, M. and Jamshidi, A., 2021. Occurrence, pathotypes, and antimicrobial resistance profiles of diarrheagenic *Escherichia coli* strains in animal source food products from public markets in Mashhad, Iran. *Food Control*, 121, p.107640.

Girard, M. *et al.* (2020) ‘Chestnut extract but not sodium salicylate decreases the severity of diarrhea and enterotoxigenic *Escherichia coli* F4 shedding in artificially infected piglets’, *PLoS One*, 15(2), p. e0214267. https://doi.org/10.1371/journal.pone.0214267.

Paton, A.W. and Paton, J.C. (1998) ‘Detection and characterization of Shiga toxigenic *Escherichia coli* by using multiplex PCR assays for *stx1*, *stx2*, *eaeA*, enterohemorrhagic *E. coli* *hlyA*, *rfbO111*, and *rfbO157*’, *Journal of clinical microbiology*, 36(2), pp. 598–602.

Rúgeles, L.C., Bai, J., Martínez, A.J., Vanegas, M.C. and Gómez-Duarte, O.G., 2010. Molecular characterization of diarrheagenic *Escherichia coli* strains from stools samples and food products in Colombia. *International Journal of Food Microbiology*, 138(3), pp.282-286.

Sobhy, N.M. et al. (2020) ‘Virulence factors and antibiograms of *Escherichia coli* isolated from diarrheic calves of Egyptian cattle and water buffaloes’, PLoS One, 15(5), p.e0232890. <https://doi.org/10.1371/journal.pone.0232890>.

Talukdar, P.K., Rahman, M., Rahman, M., Nabi, A., Islam, Z., Hoque, M.M., Endtz, H.P. and Islam, M.A., 2013. Antimicrobial resistance, virulence factors and genetic diversity of *Escherichia coli* isolates from household water supply in Dhaka, Bangladesh. *PLoS One*, 8(4), p.e61090.

Wang, R.F., Cao, W.W. and Cerniglia, C.E., 1996. PCR detection and quantitation of predominant anaerobic bacteria in human and animal fecal samples. *Applied and Environmental Microbiology*, 62(4), pp.1242-1247.
